# Glucose-tolerant β-glucosidase retrieved from a Kusaya gravy metagenome

**DOI:** 10.3389/fmicb.2015.00548

**Published:** 2015-06-16

**Authors:** Taku Uchiyama, Katusro Yaoi, Kentaro Miyazaki

**Affiliations:** ^1^Bioproduction Research Institute, National Institute of Advanced Industrial Science and Technology TsukubaIbaraki, Japan; ^2^Department of Computational Biology and Medical Sciences, Graduate School of Frontier Sciences, The University of TokyoKashiwa, Japan

**Keywords:** β-glucosidase, cellulosic biomass, enzymatic saccharification, metagenome, substrate inhibition, product inhibition

## Abstract

β-glucosidases (BGLs) hydrolyze cello-oligosaccharides to glucose and play a crucial role in the enzymatic saccharification of cellulosic biomass. Despite their significance for the production of glucose, most identified BGLs are commonly inhibited by low (∼mM) concentrations of glucose. Therefore, BGLs that are insensitive to glucose inhibition have great biotechnological merit. We applied a metagenomic approach to screen for such rare glucose-tolerant BGLs. A metagenomic library was created in *Escherichia coli* (∼10,000 colonies) and grown on LB agar plates containing 5-bromo-4-chloro-3-indolyl-β-D-glucoside, yielding 828 positive (blue) colonies. These were then arrayed in 96-well plates, grown in LB, and secondarily screened for activity in the presence of 10% (w/v) glucose. Seven glucose-tolerant clones were identified, each of which contained a single *bgl* gene. The genes were classified into two groups, differing by two nucleotides. The deduced amino acid sequences of these genes were identical (452 aa) and found to belong to the glycosyl hydrolase family 1. The recombinant protein (Ks5A7) was overproduced in *E. coli* as a C-terminal 6 × His-tagged protein and purified to apparent homogeneity. The molecular mass of the purified Ks5A7 was determined to be 54 kDa by SDS-PAGE, and 160 kDa by gel filtration analysis. The enzyme was optimally active at 45°C and pH 5.0–6.5 and retained full or 1.5–2-fold enhanced activity in the presence of 0.1–0.5 M glucose. It had a low K_M_ (78 μM with *p*-nitrophenyl β-D-glucoside; 0.36 mM with cellobiose) and high *V*_max_ (91 μmol min^-1^ mg^-1^ with *p*-nitrophenyl β-D-glucoside; 155 μmol min^-1^ mg^-1^ with cellobiose) among known glucose-tolerant BGLs and was free from substrate (0.1 M cellobiose) inhibition. The efficient use of Ks5A7 in conjunction with *Trichoderma reesei* cellulases in enzymatic saccharification of alkaline-treated rice straw was demonstrated by increased production of glucose.

## Introduction

Cellulose, the most abundant component of biomass on earth, is a linear polymer of D-glucose linked by β-1,4-glucosidic bonds. Because of the increasing demand for energy and the continuous depletion of fossil fuels, the production of bio-energy and bio-based products from cellulosic biomass is one of the biggest challenges in biotechnology. The breakdown of cellulosic biomass to glucose involves physical–chemical treatment followed by enzymatic saccharification of the raw material. The enzymatic process involves the synergistic actions of four classes of enzymes: (i) endo-β-1,4-glucanase (EC 3.2.1.4); (ii) exo-cellobiohydrolase (EC 3.2.1.91); (iii) copper-dependent lytic polysaccharide monooxygenase; and (iv) β-glucosidase (EC 3.2.1.21, BGL). Endo-glucanase and exo-cellobiohydrolase act on cellulose to produce cellobiose, which often inhibits the activities of the enzymes that catalyze its production (Coughlan, 1985; Kadam and Demain, 1989; Watanabe et al., 1992). β-glucosidases (BGLs) act on cellobiose (and cello-oligosaccharides) to produce glucose; this can reduce the inhibitory effect of cellobiose on endo-glucanase and exo-cellobiohydrolase (Xin et al., 1993; Saha et al., 1994). However, most of the microbial BGLs known to date are highly sensitive to glucose (Gueguen et al., 1995; Saha et al., 1995). Furthermore, BGLs are also inhibited by their substrate, cellobiose (Woodward and Wiseman, 1982; Schmid and Wandrey, 1987). Thus, the development of BGLs that are insensitive to glucose and cellobiose inhibition will have a significant impact on the enzymatic saccharification of cellulosic biomass and will accelerate the entire process of cellulose breakdown.

To date, several glucose-tolerant BGLs have been identified in insects (Uchima et al., 2011), fungi (Saha and Bothast, 1996; Yan and Lin, 1997; Riou et al., 1998; Decker et al., 2001; Zanoelo et al., 2004; Souza et al., 2014), bacteria (Pérez-Pons et al., 1995), and metagenomes (Fang et al., 2010; Biver et al., 2014). Recently, we have identified a glucose-tolerant BGL (Td2F2) in a wood compost metagenomic library (Uchiyama et al., 2013). Td2F2 has a unique property in that its activity is not reduced by glucose but is stimulated in the presence of high concentrations of glucose (0.1 M or higher). The basis for this unique property is its high transglycosylation activity. The tolerance to glucose and high transglycosylation activity of Td2F2 will be strongly advantageous when it is used in the enzymatic saccharification of cellulose as well as the enzymatic synthesis of stereo- and regio-specific glycosides.

To identify other potentially useful BGLs, we screened a metagenomic library of Kusaya (a Japanese traditional fermentation product made from fish) gravy as a source for genomes. The library was constructed in *Escherichia coli*, which was first screened for BGL activity in the absence of glucose. Positive clones were then screened in the presence of glucose. As a result of this screen, we successfully obtained a glucose-tolerant BGL, which we named Ks5A7. The gene encoding Ks5A7 was overexpressed in *E. coli*, and the recombinant enzyme was characterized. We applied Ks5A7 to the saccharification of alkaline-treated rice straw, in combination with fungal cellulases from *Trichoderma reesei*, to demonstrate its efficiency for enhancing the production of glucose.

## Materials and Methods

### Reagents

Restriction endonucleases, DNA ligase, and DNA polymerase were purchased from Takara Bio (Shiga, Japan). The QIAquick Kit was obtained from Qiagen (Hilden, Germany). 5-Bromo-4-chloro-3-indolyl-β-D-glucoside (X-glc) was purchased from Rose Scientific (Edmonton, AB, Canada). p-Nitrophenyl (pNP) α-D-galactopyranoside, pNP α-D-glucopyranoside, and pNP β-D-xylopyranoside were purchased from Nacalai (Kyoto, Japan). pNP α-D-mannopyranoside was purchased from Senn Chemicals (Zürich, Switzerland). The following chemicals were purchased from Sigma (St. Louis, MO, USA): avicel, pNP α-L-arabinofuranoside, pNP α-L-arabinopyranoside, pNP β-L-arabinopyranoside, pNP α-L-fucopyranoside, pNP β-D-fucopyranoside (pNPFuc), pNP β-D-galactopyranoside, pNP β-D-glucopyranoside (pNPGlc), pNP β-D-mannopyranoside, pNP N-acetyl-β-D-glucosaminide, pNP α-L-rhamnopyranoside, pNP α-D-xylopyranoside, and pNP β-D-cellobioside, sophorose, nigerose, maltose, isomaltose, lactose, and salicin. Cello-origosaccharides and laminaribiose were purchased from Seikagaku Kogyo (Tokyo, Japan). Gentiobiose was purchased from Tokyo Chemical Industry (Tokyo, Japan).

### Library Construction and Screening for BGLs

Kusaya gravy was sampled at Niijima Island, Tokyo, Japan in May, 2007. The metagenome was purified, fragmented by partial digestion with *Sau*3AI, and ligated into a p18GFP vector at the *Bam*HI site, as described previously (Uchiyama and Watanabe, 2008). *E. coli* DH10B cells were transformed with the ligation mixture and grown at 37°C overnight on LB agar plates containing 100 μg mL^-1^ ampicillin (Amp) to yield ∼380,000 colonies. The colonies were scraped from the plates, mixed well, appropriately diluted, and regrown on LB agar plates containing 100 μg mL^-1^ Amp, 10 μM isopropyl-β-D-thio-galactopyranoside (IPTG), and 20 μg mL^-1^ X-glc. Approximately 10,000 colonies appeared on the plates; colonies that turned blue in color after prolonged incubation at 4°C for 3 weeks were selected and arrayed in a 96-well format.

### Screening for Glucose-Tolerant BGLs

Blue *E. coli* colonies arrayed in 96-well plates were grown in 800 μL of LB liquid medium containing 100 μg mL^-1^ Amp, 10 μM IPTG at 37°C overnight with vigorous agitation (1,000 rpm) in a Taitec (Saitama, Japan) MBR-420FL shaker. Cultures were then transferred to three 96-well plates (200 μL each, with the remaining 200 μL reserved for stock), pelleted by centrifugation (3,220 × *g*, 15 min, 4°C), and the supernatant discarded. Cells were resuspended in 0.1 M sodium phosphate buffer, pH 6.0, containing 1 mM pNPGlc and 0 or 10% (w/v) glucose, and incubated at 37°C with agitation (1,000 rpm). After 48 h, cells were pelleted by centrifugation (3,220 × *g*, 15 min, 4°C), and 50 μL of the supernatants were transferred to fresh 96-well plates; 100 μL of 0.1 M Na_2_CO_3_ was added to each well, and absorbance at 405 nm was read using a Molecular Devices (Sunnyvale, CA, USA) plate reader (VersaMax).

### DNA Sequencing and Sequence Data Analysis

A shotgun DNA library was produced using plasmids partially digested with *Alu*I. The products were separated by agarose gel electrophoresis and fragments 1–3 kb in length were gel-purified and cloned into a suicide vector pre-digested with *Sma*I (Miyazaki, 2010). The DNA sequences of the cloned fragments were determined from one end of the vector, flanked by the *Sma*I site, by the Sanger method. A sequence similarity search was performed using BLAST software (Altschul et al., 1997) and the National Center for Biotechnology Information (NCBI) database.

### Production and Purification of Recombinant Ks5A7

To remove two *Nde*I sites encoded in the *ks5a7* gene, two rounds of QuikChange-based site-directed mutagenesis (Weiner et al., 1994) were performed using sets of primers xNdeI-1+ and xNdeI-1-, followed by xNdeI-2+ and xNdeI-2- (**Table 1**). After removing the two *Nde*I sites, the *ks5a7* gene was amplified by PCR using forward (Ks5A7Fwd) and reverse (Ks5A7Rev) primers (**Table 1**). The amplicon (1.4 kbp) was gel-purified, digested with *Nde*I and *Xho*I, and cloned into the same sites of the pET29b (+) vector to fuse a 6 × His-tag to the C-terminus of the recombinant protein. The expression plasmid was introduced into *E. coli* Rosetta (DE3) and grown on LB agar plates containing 50 μg mL^-1^ kanamycin and 34 μg mL^-1^ chloramphenicol. A single colony was selected and grown in 1 L of Overnight Express Instant LB Medium (Novagen, Madison, WI, USA) containing 50 μg mL^-1^ kanamycin and 34 μg mL^-1^ chloramphenicol at 30°C with agitation (200 rpm). After 18 h, cells were collected by centrifugation (5,000 × *g*, 10 min, 4°C) and resuspended in 100 mL of BugBuster (Novagen) and Benzonase (Novagen). After gentle agitation at room temperature for 30 min, debris was removed by centrifugation (15,000 × *g*, 20 min, 4°C). The supernatant was then loaded onto a Ni-NTA column (5 mL; Qiagen, Hilden, Germany) pre-equilibrated with 20 mM sodium phosphate buffer (pH 7.4) containing 0.5 M NaCl. After washing the column with 100 mL of 20 mM sodium buffer (pH 7.4) containing 0.5 M NaCl, the column was further washed with 100 mL of 20 mM sodium phosphate buffer (pH 7.4) containing 0.5 M NaCl and 25 mM imidazole. Bound proteins were then eluted with a linear gradient of imidazole from 25 to 500 mM in 20 mM sodium phosphate buffer (pH 7.4) containing 0.5 M NaCl, over a total volume of 100 mL. Active fractions were combined and buffer-exchanged to 20 mM sodium phosphate (pH 7.4) containing 50 mM NaCl using an Amicon Ultra-15. The concentration of Ks5A7 was determined based on the molecular coefficient of 117,035 M^-1^ cm^-1^ at 280 nm. Calculations were performed using the ProtParam tool at http://www.expasy.ch/tools/protparam.html (Gasteiger et al., 2005).

**Table 1:** Oligonucleotide primers used in this study.

Primer sequence	
xNdeI-1+	5′- ATGATATTGTTCCATAcGTTACTCTTTTTCACTGG -3′
xNdeI-1–	5′- CCAGTGAAAAAGAGTAACgTATGGAACAATATCAT -3′
xNdeI-2+	5′- TTCTTGACTTAAATGATGCATAcGTCTGGTCTGTTTCATT -3′
xNdeI-2–	5′- AATGAAACAGACCAGACgTATGCATCATTTAAGTCAAGAA -3′
Ks5A7Fwd	5′- AAAAACATATGATGAAATTTAATGAAAACTTTGTTTGGGGT -3′
Ks5A7Rev	5′- TTTTTCTCGAGTAGGTTCTCACCATTTTCTTCAATA -3′
E163Q+	5′- AAATACATTATGACATTTAATcAACCTCAGTGCACAATT -3′
E163Q–	5′- AATTGTGCACTGAGGTTgATTAAATGTCATAATGTATTT -3′
E357Q+	5′- ACCTACCTTTTTATATAACTcAAAACGGCCTTGC -3′
E357Q–	5′- GCAAGGCCGTTTTgAGTTATATAAAAAGGTAGGT -3′

### Construction, Expression, and Protein Purification of E163Q and E3570Q Variants of Ks5A7

Site-directed mutagenesis was carried out following the QuikChange protocol (Weiner et al., 1994). For E163Q, a set of complementary primers (E163Q+ and E163Q–, **Table 1**) was used. For E357Q, a set of complementary primers (E357Q+ and E3457–, **Table 1**) was used. Gene expression and protein purification were carried out in the same manner as for the wild type.

### Molecular Mass

Polyacrylamide gel electrophoresis was performed under denaturing conditions using a DRC XV Pantera gel (7.5–15% [w/v] gradient polyacrylamide) in a Tris-Glycine buffer system containing 0.1% (w/v) sodium dodecylsulfate. Samples were heat-treated at 95°C for 5 min with 2-mercaptoethanol and 0.1% (w/v) sodium dodecylsulfate prior to electrophoresis. Gel filtration analysis was carried out using a GE Healthcare column (Superose 6 10/300 GL, 1 cm × 30 cm) in 20 mM Tris-HCl (pH 7.0) containing 0.2 M NaCl and 10 mM dithiothreitol at a flow rate of 0.5 mL min^-1^. The molecular weight standards were thyroglobulin (670 kDa), bovine γglobulin (158 kDa), chicken ovalbumin (44 kDa), equine myoglobin (17 kDa), and vitamin B12 (1.35 kDa).

### Enzyme Assays

Enzyme activity was routinely assayed in a 85-μL reaction mixture containing McIlvaine buffer (pH 5.5; McIlvaine, 1921), 5 mM pNPGlc, and 1.0 ng μL^-1^ enzyme. After 5 min of incubation at 45°C, the reaction was stopped by incubation at 95°C for 3 min; 85 μL of 0.2 M Na_2_CO_3_ was added to the mixture, and the levels of liberated *p*-nitrophenol (pNP) were determined at 405 nm using a Molecular Devices plate reader (VersaMax). Optimal reaction temperature and pH were determined by changing the assay temperature or buffers in the presence of 5 mM pNPGlc and 1.0 ng μL^-1^ enzyme. Inhibition of pNPGlc hydrolysis by glucose was tested in a 85-μL reaction mixture containing 5–20 mM pNPGlc, McIlvaine buffer (pH 5.5), 1.0 ng μL^-1^ enzyme, and varied concentrations of glucose (0–0.5 M). Kinetic constants were determined at 45°C from the initial rate of activity. The reaction was performed for 5 min and stopped by incubation at 95°C for 3 min. For pNPGlc and pNPFuc, the assay was performed in a 85-μL reaction mixture containing McIlvaine buffer (pH 5.5), 0.0156–0.5 mM substrate, and 0.1 ng μL^-1^ enzyme; 85 μL of 0.2 M Na_2_CO_3_ was added to the mixture, and levels of liberated pNP were determined.

The enzyme activity with respect to oligosaccharide substrates was determined in a 50-μL reaction mixture containing McIlvaine buffer (pH 5.5), 1.0 mg mL^-1^ substrate, and 0.1 ng μL^-1^ enzyme. The reaction was stopped by heating the sample to 98°C for 5 min. The concentration of released glucose was determined using an Invitrogen Amplex Red glucose/glucose oxidase assay kit, according to the manufacturer’s instructions. The kinetic constants for cello-oligosaccharides were determined in a 50-μL reaction mixture containing McIlvaine buffer (pH 5.5), 0.0625–4 mM substrate, and 0.1 ng μL^-1^ enzyme. The kinetic constants, K_M_ and k_cat_, were calculated by non-linear regression with the Michaelis–Menten equation using GraphPad PRISM Version 6.0 (GraphPad Software).

### Saccharification of Alkaline-Treated Rice Straw

Alkaline-treated rice straw was prepared by incubation in 0.5% [w/v] NaOH at 100°C for 5 min as described previously (Kawai et al., 2012), which was purchased from Japan Bioindustry Association. *T. reesei* strain PC-3-7 (ATCC 66589) were purchased from American Type Culture Collection (ATCC). For preparation of crude cellulases from the *T. reesei* strain PC-3-7, the fungus was cultivated on potato dextrose agar and 10^7^ conidia were collected and inoculated into 50 mL of basal medium (Kawamori et al., 1986) containing 1 % (w/v) avicel. The inoculum was cultivated for 1 week at 28°C, 220 rpm. After cultivation, the culture was centrifuged at 8,000 × *g* for 20 min at 4°C, and the supernatant was filtered. The resulting filtrate was used as the crude cellulases.

The concentration of the crude cellulases was determined using a Quick Start Bradford Dye Reagent (Bio-Rad Laboratories, Hercles, CA, USA) with bovine γ-globulin as the standard. Saccharification of alkaline-treated rice straw was performed in a hermetically closed 20-mL plastic bottle at 50°C, with shaking at 150 rpm. The reaction medium contained 50 mg mL^-1^ alkaline-treated rice straw, 100 mM sodium acetate buffer pH 5.0, 0.2 mg mL^-1^ sodium azide, and 150 μg mL^-1^ of crude cellulases. BGL (Ks5A7 or Td2F2) was added to a concentration of 5 μg mL^-1^. After the reaction, the supernatants were boiled for 5 min, and the production of glucose and cellobiose was measured by HPLC following the method described previously (Kawai et al., 2012). Preparation of Td2F2 was as described previously (Uchiyama et al., 2013).

### Nucleotide Sequence Accession Numbers

The nucleotide sequence for Ks5A7 has been deposited in GenBank/EMBL/DDBJ under the accession number HV348683.

## Results and Discussion

### Screening for BGL in a Metagenomic Library of Kusaya Gravy

A metagenomic library was constructed in *E. coli* using Kusaya gravy, a traditional Japanese fermentation food product of dried fish, as a source of the metagenome. The library containing ∼380,000 clones included insert fragments ranging from 5 to 20 kbp in length. A portion of the library (∼10,000 clones) was used to screen for BGL by growing on LB agar plates containing X-glc as a substrate. Although overnight cultivation generated very few positive (i.e., blue) colonies, prolonged incubation at 4°C gradually increased the number of positive colonies, yielding ∼1,000 blue colonies after 3 weeks. The positive colonies were then streaked onto LB agar plates containing X-glc for single isolation, yielding 828 clones in total.

### Screening for Glucose-Tolerant BGLs

The clones initially identified as positive were arrayed in a 96-well format. Clones were grown in LB, and whole cells were used to determine activity in the presence and absence of 10% (w/v) glucose. Although the majority of clones exhibited no activity in the presence of 10% (w/v) glucose, seven (5A7, 5B6, 5F2, 6C8, 7F9, 9B4, and 10H11) retained >20% activity relative to the glucose-free condition. DNA sequencing was performed from one end of the plasmids, revealing that three clones (7F9, 9B4, and 10H11) had identical insert fragments.

### DNA Sequencing of Glucose-Tolerant BGLs

Plasmids were purified from the five different clones: 5A7, 5B6, 5F2, 6C8, and 7F9. For each plasmid, a total of 96 shotgun clones were analyzed. Although no complete *bgl* gene was obtained from the partially determined nucleotide sequences, the results suggested that the clones carried *bgl* genes with high identity. We then synthesized a set of PCR primers to amplify the *bgl* gene from the five plasmids. All five clones produced a 1.4-kbp amplicon. DNA sequencing of the fragments revealed that the five *bgl* genes could be classified into two groups, differing by only two nucleotide substitutions. The deduced amino acid sequences were identical, and the gene obtained from clone 5A7 was used for subsequent studies.

The *bgl* gene *ks5a7* contained 1,359 bp, with a GC content of 32.3%. The predicted ATG initiation codon was preceded by a possible ribosomal binding site, 5′-AAGAGGA-3′. The deduced amino acid sequence contained 452 amino acids and had a calculated molecular mass of 52,509 Da.

Using BLAST-P^1^, we found that Ks5A7 was highly similar to enzymes belonging to the glycoside hydrolase family 1 (GH1) of the carbohydrate-active enzyme classification database (Lombard et al., 2014)^2^. Ks5A7 exhibited the highest identity (57%) with a putative BGL from *Clostridiales bacterium* oral taxon 876 and a 55% identity with a putative BGL from *Clostridium hathewayi* DSM13479. When compared with functionally characterized BGLs, the Ks5A7 showed the highest (46%) identity with that of *Thermotoga neapolitana* (Yernool et al., 2000; Park et al., 2005).

### Overproduction of Ks5A7

Ks5A7 was produced as a C-terminal 6 × His-tagged protein using a pET system (Studier and Moffatt, 1986). Two *E. coli* strains, Rosetta (DE3), and BL21 (DE3), were tested as a host. Approximately 2.5-fold higher activity was obtained from the cell extract prepared from Rosetta (DE3) compared with that from BL21 (DE3). Ks5A7 contained a high rate of rare codons (52 of a total 452 amino acids). Of particular note, all 17 Arg residues were encoded by rare codons: 14 AGA, 2 CGA, and 1 AGG. Because Rosetta (DE3) carries a plasmid containing seven genes for rare tRNA codons, including those for AGA, and AGG, the low production level in BL21 (DE3) might have been improved in Rosetta (DE3) as a result of the supply of rare tRNAs. In terms of temperature, in Rosetta (DE3), the activity was 10-fold higher at 30°C than at 37°C.

Expressed recombinant protein was readily purified to homogeneity using a Ni-NTA column. A large quantity of purified enzyme was recovered, with a typical final yield of 70 mg L^-1^ culture, representing a 30% yield.

### General Properties of Ks5A7

**FIGURE 1:**
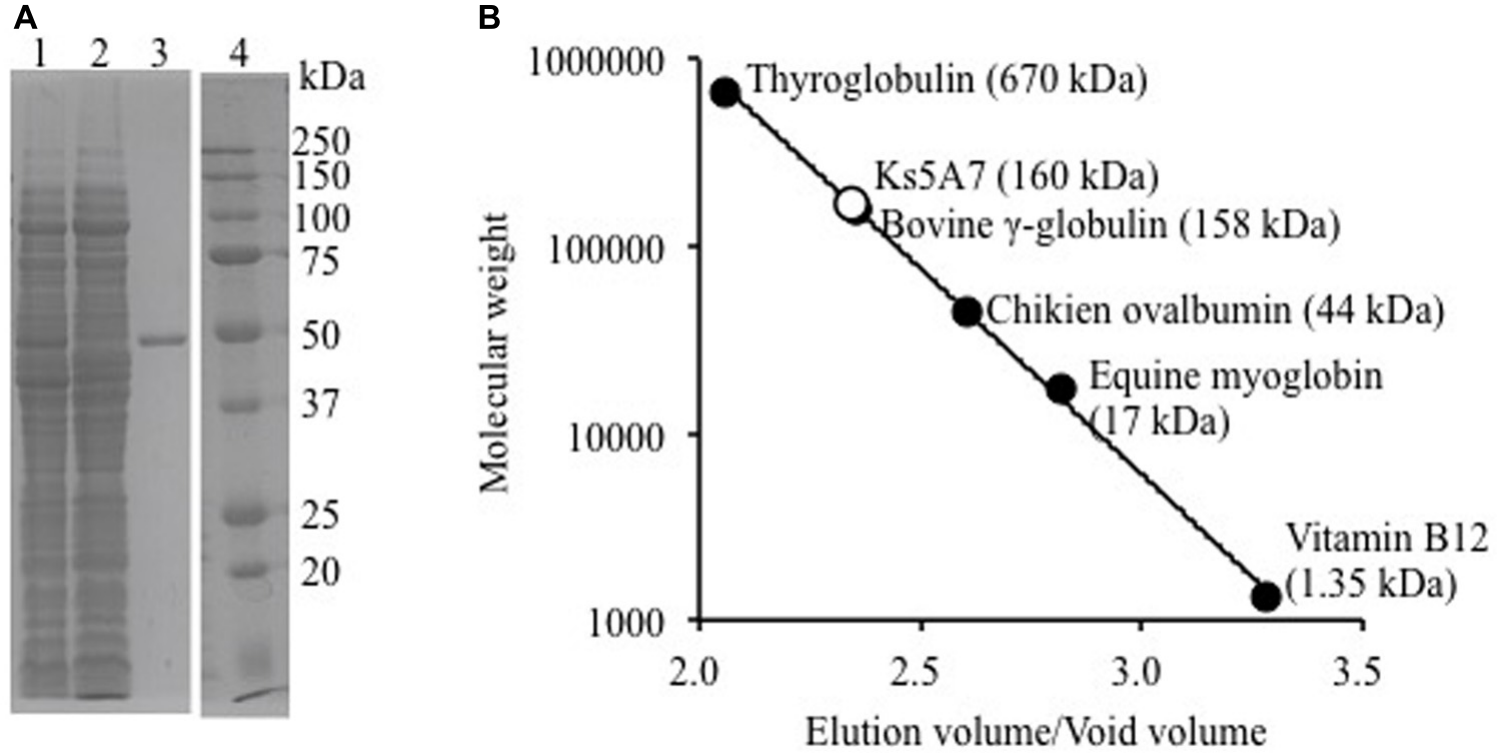
**Molecular mass analysis of recombinant Ks5A7. (A)** SDS-PAGE. Lane 1, soluble protein fraction; lane 2, flow-through from Ni-NTA column; lane 3, purified Ks5A7; lane 4, molecular markers. Ks5A7 migrated at ∼50 kDa. **(B)** Gel filtration. Symbols: solid circles, molecular mass of protein markers; open circle, Ks5A7. Ks5A7 was eluted at ∼160 kDa.

Purified recombinant Ks5A7 had a molecular mass of ∼50 kDa according to SDS-PAGE (**Figure 1A**), which is in agreement with the mass calculated from the deduced amino acid sequence (53,573 Da). The molecular mass of the native structure of Ks5A7 was determined by gel filtration column chromatography (**Figure 1B**). Ks5A7 was eluted at the 160 kDa position, suggestive of multimeric states (trimer or tetramer).

**FIGURE 2:**
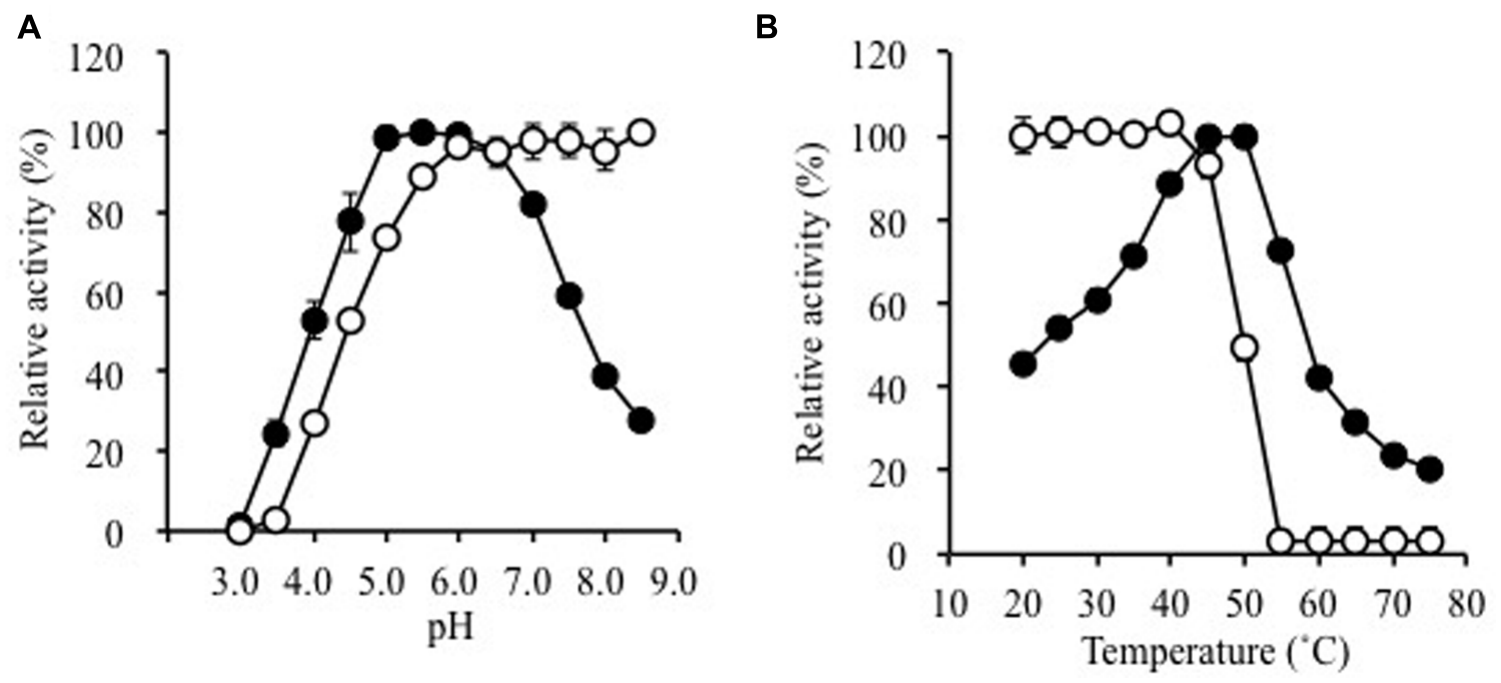
**Effects of (A) pH and (B) temperature on the activity (solid circles) and stability (open circles) of purified Ks5A7**. With regard to the pH-dependence of stability, the enzyme was incubated for 30 min at various pH values. In terms of the pH-dependence of activity, the enzyme was assayed at various pH values by the standard assay method. To address the temperature-dependence of stability, the enzyme was incubated for 10 min at various temperatures. In terms of the temperature-dependence of activity, the enzyme was assayed at various temperatures by the standard assay method. Error bars, SD. *N* = 3.

The pH-stability and pH-dependence of activity are illustrated in **Figure 2A**. The enzyme was fairly stable at pH 5.5–8.5 (30 min at 25°C). It was optimally active between pH 5.0 and 6.0 (specific activity, 49.1 ± 0.4 μmol min^-1^ mg^-1^) with ∼80% activity at pH 4.5 and 7.0, respectively. The effects of temperature on stability and activity are shown in **Figure 2B**. The enzyme was inactivated upon incubation at 55°C for 10 min. Maximal activity was observed at 50°C in a 5-min assay (specific activity, 58.4 ± 1.4 μmol min^-1^ mg^-1^).

On the basis of similarity to the known GH1 family BGLs, it has been inferred that E163 and E357 function as an acid-base catalyst and nucleophile, respectively (Withers et al., 1990; Wang et al., 1995). They were individually substituted to glutamine, and the resultant mutant enzymes were characterized. No activity was observed when 5 mM pNPGlc was used for both enzymes (data not shown), suggesting the same roles for these residues in catalysis as observed in other GH1 BGLs.

### Activity with *p*-Nitrophenyl Substrates and Oligosaccharides

**FIGURE 3:**
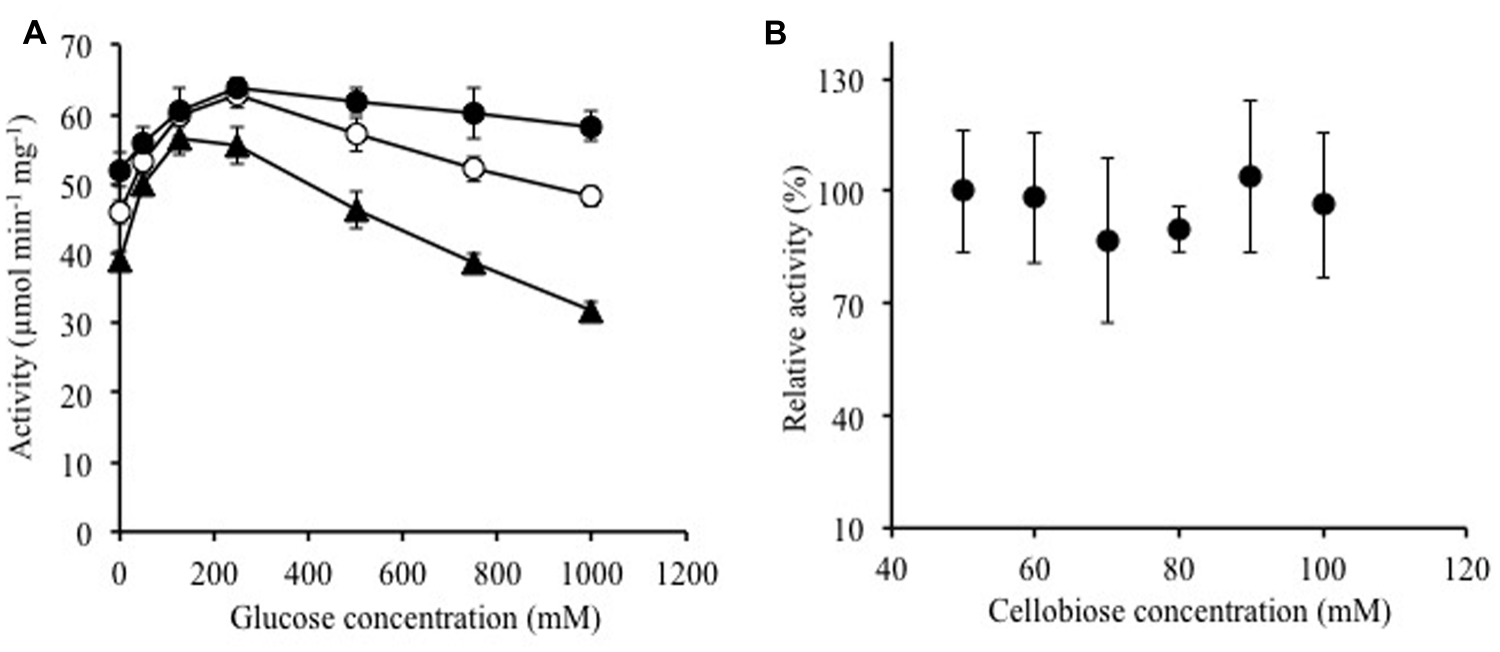
**Effects of (A) glucose and (B) cellobiose on enzymatic activity of Ks5A7. (A)** Activities were determined in the presence of various concentrations of glucose using 5 (solid triangles), 10 (open circles), or 20 mM of pNPGlc (solid circles) at 45°C. **(B)** Activities were determined in the presence of various concentrations of cellobiose at 45°C. The specific activity for cellobiose was measured by evaluating the glucose concentration. The activity for 50 mM cellobiose was taken to be 100%. The specific activity was 131.9 ± 21 μmol min^-1^ mg^-1^. Error bars, SD. *N* = 3.

The substrate specificity of Ks5A7 was characterized using a fixed concentration (5 mM) of various *p*-nitrophenyl substrates and oligosaccharides. For *p*-nitrophenyl substrates, the enzyme showed the highest activity for pNPFuc, followed by pNPGlc (**Table 2**). Dual pNPFuc and pNPGlc activities have been reported for a BGL enzyme from *Bifidobacterium breve* (Nunoura et al., 1996a,b). However, the activity of *Bifidobacterium* BGL lost 30% of its original activity in the presence of 0.1 M glucose, whereas Ks5A7 displayed 150% activity under the same conditions (see below, **Figure 3A**). Both enzymes belong to the GH1 family but share only 37% of their amino acid sequence identity. Therefore, these two enzymes are distinct, and the basis for the dual pNPFuc/pNPGlu activities remains unknown.

**Table 2:** Substrate specificity of the recombinant Ks5A7.

Substrate^a^	Linkage of glycosyl group	Relative activity (%)
Chromogenic substrates
pNPGlc	β-Glucose	100 ± 9.7^b^
pNPFuc	β-Fucose	164 ± 12
*p*-Nitrophenyl-β-D-galactopyranoside	β-Galactose	64.5 ± 5.5
*p*-Nitrophenyl-β-D-xylopyranoside	β-Xylose	1.05 ± 0.0
*p*-Nitrophenyl-β-D-cellobioside	β-Cellobiose	7.88 ± 0.0
*p*-Nitrophenyl-β-D-lactopyranoside	β-Lactose	46.8 ± 5.5
*p*-Nitrophenyl-α-D-glucopyranoside	α-Glucose	2.54 ± 0.0
*p*-Nitrophenyl-α-L-arabinopyranoside	α-Alabinose	3.31 ± 0.0
Oligosaccharides
Cellobiose	β(1,4)Glucose	100 ± 8.4^c^
Cellotriose	β(1,4)Glucose	116 ± 7.0
Cellotetraose	β(1,4)Glucose	121 ± 21
Cellopentaose	β(1,4)Glucose	90.1 ± 8.8
Laminaribiose	β(1,3)Glucose	112 ± 0.8
Sophorose	β(1,2)Glucose	65.5 ± 9.7
Salicin	β(1,4)Glucose	59.1 ± 3.8

*^a^No activity was detected with p-nitrophenyl-β-D-mannopyranoside, p-nitrophenyl- N-acetyl-β-D-glucosaminide, p-nitrophenyl-β-L-arabinopyranoside, p-nitrophenyl-α-D-galactopyranoside, p-nitrophenyl-α-D-xylopyranoside, p-nitrophenyl-α-L-fucopyranoside, p-nitrophenyl-α-L-arabinofuranoside, and p-nitrophenyl-α-L-rhamnopyranoside, oligosaccharides, such as gentiobiose, nigerose, maltose, isomaltose, and lactose*.

*^b^The specific activity of Ks5A7 for pNPGlc was 53.9 ± 5.2 μmol min^-1^ mg^-1^, by measuring the release of pNP*.

*^c^The specific activity of Ks5A7 for cellobiose was 170 ± 20 μmol min^-1^ mg^-1^, by measuring the release of glucose*.

As shown in **Table 2**, Ks5A7 was found to possess enzyme activity for cello-oligosaccharides from cellobiose to cellopentaose. Ks5A7 hydrolyzed a range of β*-*linked glycosides including β(1,2), β(1,3), and β(1,4) but not β(1,6). No activity was detected for the oligosaccharides with α-linkages.

### Kinetic Constants of Ks5A7

The steady-state kinetic constants of Ks5A7 for pNPGlc, pNPFuc, and cello-oligosaccharides are shown in **Table 3**. The K_M_ for pNPFuc was higher (0.152 mM) than that for pNPGlc, but the V_max_ (137 μmol min^-1^ mg^-1^) was also higher for pNPFuc, resulting in similar overall catalytic efficiency (k_cat_/K_M_) for the two substrates. Compared with other known glucose-tolerant BGLs (Pérez-Pons et al., 1995; Saha and Bothast, 1996; Yan and Lin, 1997; Riou et al., 1998; Decker et al., 2001; Zanoelo et al., 2004; Fang et al., 2010; Uchima et al., 2011; Uchiyama et al., 2013; Biver et al., 2014; Souza et al., 2014), the K_M_ of Ks5A7 for pNPGlc was the lowest (0.078 mM) and the V_max_ was relatively high (90.8 μmol min^-1^ mg^-1^).

**Table 3:** Steady-state kinetic constants of the recombinant Ks5A7.

Substrate	K_M_ (mM)	V_max_ (μmol min^-1^ mg^-1^)	k_cat_ (s^-1^)	k_cat_/K_M_ (s^-1^ mM^-1^)
Aryl-β-glycosides
pNPGlc	0.078 ± 0.002	90.8 ± 0.7	81.0 ± 0.6	1045
pNPFuc	0.152 ± 0.004	137 ± 0.2	122 ± 1.2	803
Oligosaccharides
Cellobiose	0.358 ± 0.055	155 ± 8.3	138 ± 7.4	386
Cellotriose	0.163 ± 0.016	111 ± 3.2	99.1 ± 2.8	610
Cellotetraose	0.160 ± 0.012	114 ± 2.5	101 ± 2.2	634
Cellopentaose	0.132 ± 0.011	112 ± 2.5	99.7 ± 2.2	753

For cello-oligosaccharides, the K_M_ value was highest with cellobiose as a substrate, and it gradually decreased as the chain length increased, suggesting that the active site include subsites that accommodate the oligosaccharides. The absence of glucose inhibition is presumably because the small glucose molecule cannot efficiently bind to the active site. The V_max_ value was slightly higher with cellobiose than with other cello-oligosaccharides. The overall reaction efficiency was highest with cellopentaose. The time-course analysis of cellopentaose hydrolysis by HPLC revealed that the only products were cellotetraose and glucose, indicating that glucose was liberated from cellopentaose, and confirming the exo-type of activity of Ks5A7 (data not shown). Compared with known glucose-tolerant BGLs (Pérez-Pons et al., 1995; Saha and Bothast, 1996; Riou et al., 1998; Zanoelo et al., 2004; Fang et al., 2010; Uchiyama et al., 2013; Biver et al., 2014; Souza et al., 2014), Ks5A7 had the lowest K_M_ (0.36 mM) for cellobiose and a relatively high V_max_ (155 μmol min^-1^ mg^-1^).

### Effect of Solvents, Metal Ions, and Chelating and Reducing Agents

The effects of various regents and metal cations were examined (**Table 4**); 10% (v/v) ethanol did not affect the activity, whereas 25% (v/v) ethanol reduced the activity to 44%. The addition of 10% or 25% (v/v) DMSO reduced enzyme activity. Among the metal ions tested (1 mM fixed concentration), significant inactivation was observed with CuCl_2_ and ZnCl_2_, whereas more than 70% of the activity remained in the presence of AlCl_3_, CaCl_2_, CoCl_2_, FeCl_3_, MgCl_2_, MnCl_2_, and NiCl_2_. The chelating agent EDTA (10 mM) did not affect enzyme activity, suggesting that divalent cations are not involved in catalysis. The reducing agent dithiothreitol (10 mM) slightly reduced activity (to 92%), indicating that the seven cysteines in each protein (per subunit) might be involved in catalysis or structural formation.

**Table 4:** Effects of organic solvents, metal ions, and chelating agent on the enzyme activities of the recombinant Td2F2.

Reagent	Concentration	Relative activity (%)
No additive		100 ± 0.9^a^
Ethanol	10% v/v	102 ± 2.5
Ethanol	25% v/v	44.2 ± 2.2
Dimethylsulfoxide	10% v/v	85.9 ± 0.9
Dimethylsulfoxide	25% v/v	43.8 ± 1.2
AlCl_3_	1 mM	73.6 ± 2.1
CaCl_2_	1 mM	84.0 ± 0.5
CoCl_2_	1 mM	85.7 ± 1.2
CuCl_2_	1 mM	29.6 ± 0.1
FeCl_3_	1 mM	101 ± 1.4
MgCl_2_	1 mM	75.2 ± 1.9
MnCl_2_	1 mM	81.8 ± 2.7
NiCl_2_	1 mM	88.6 ± 1.6
ZnCl_2_	1 mM	43.9 ± 0.2
EDTA	10 mM	102 ± 4.0
DTT	10 mM	91.8 ± 1.1

*^a^The activity without an additional regent was taken to be 100% (specific activity 50.7 ± 0.5 μmol min^-1^ mg^-1^)*.

### Effect of Glucose and Cellobiose on Ks5A7 Activity

Ks5A7 was initially identified as a glucose-tolerant enzyme, but the screening process involved whole cells rather than extracted enzymes. Therefore, we verified that the purified enzyme also showed tolerance to glucose. As shown in **Figure 3A**, no loss of activity was observed in the tested range, 0–0.75 M, at a substrate concentration of 5 mM pNPGlc. At 1.0 M, the activity was reduced to ∼80%. To date, several BGLs that enhance activities in the presence of glucose have been identified (Pérez-Pons et al., 1995; Zanoelo et al., 2004; Fang et al., 2010; Uchima et al., 2011; Uchiyama et al., 2013; Biver et al., 2014; Souza et al., 2014). Similar to that of these enzymes, the activity of Ks5A7 was also enhanced by glucose. In the presence of 250 mM glucose (and 5 mM pNPGlc), the activity was enhanced 1.4-fold, compared with activity in the absence of glucose (**Figure 3A**). At higher concentrations of glucose, however, activity was reduced. This pattern is consistent with the sensitivity to glucose of several other glucose-activated BGLs (Pérez-Pons et al., 1995; Fang et al., 2010; Uchima et al., 2011; Souza et al., 2014).

We recently obtained another glucose-activated BGL, Td2F2, from a wood compost metagenomic library (Uchiyama et al., 2013). In the case of Td2F2, the basis for the enhanced activity in the presence of glucose is due to the strong glycosyltransferase activity (Uchiyama et al., 2013). Taking this into account, we analyzed the reaction products of Ks5A7 after incubation with 5 mM pNPGlc and 250 mM glucose. Glucose was identified as the sole product, suggesting a lack of transglycosylation activity in Ks5A7.

Using cellobiose as a substrate, we investigated substrate inhibition of the enzyme in the tested range, from 50 to 100 mM (**Figure 3B**); no substrate inhibition occurred, at least up to 100 mM cellobiose.

Product inhibition by glucose (Gueguen et al., 1995; Saha et al., 1995) and substrate inhibition by cellobiose (Woodward and Wiseman, 1982; Schmid and Wandrey, 1987) are common major problems for BGLs. Ks5A7 is resistant not only to glucose but also to cellobiose. These unique properties are ideal for cellulosic biomass degradation.

### Effect of BGLs on the Enzymatic Saccharification of Alkaline-Treated Rice Straw Hydrolysis

**FIGURE 4:**
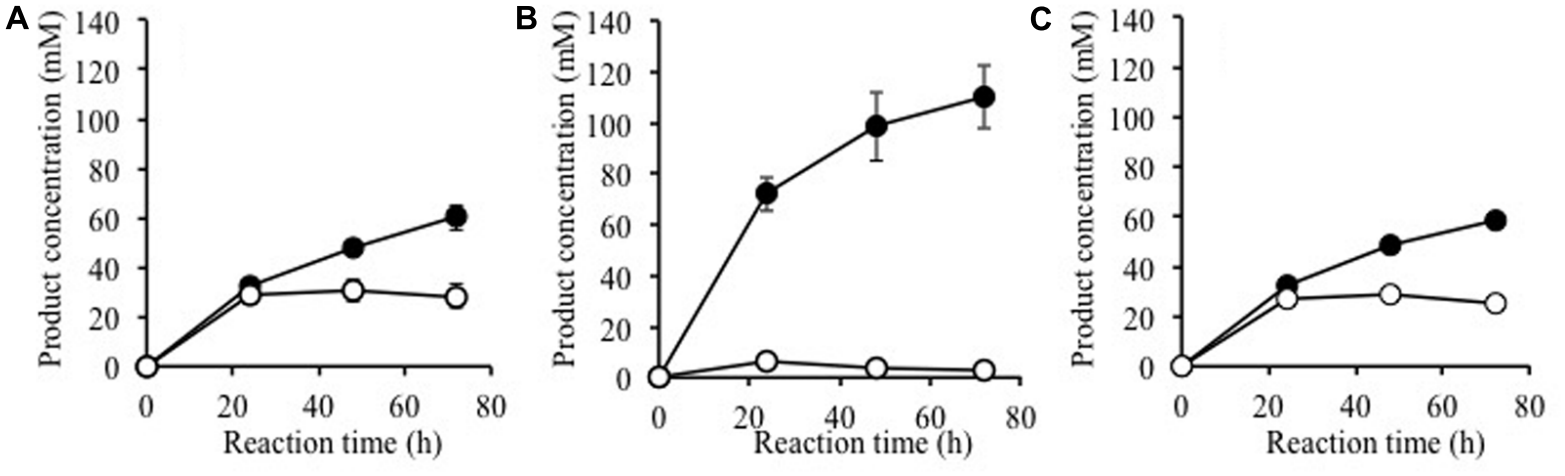
**Saccharification of alkaline-treated rice straw by the crude cellulases from *Trichoderma reesei* with β-glucosidase**. Production of glucose (solid circles) and cellobiose (open circles) are shown. The reaction was performed with **(A)** no BGL addition, **(B)** plus Ks5A7, and **(C)** plus Td2F2. Error bars, SD. *N* = 3.

Using alkaline-treated rice straw as a substrate, we investigated whether Ks5A7 (or Td2F2) would be effective for the enzymatic degradation of cellulosic materials. Cellulases from *T. reesei* PC3-7 were used as base enzymes in the reaction, to which a BGL (Ks5A7 or Td2F2) was added (**Figure 4**). Compared with the control (no BGL addition, **Figure 4A**, filled circle), a two fold increase of glucose was observed for Ks5A7 (**Figure 4B**, filled circle), which was much more effective than Td2F2 (**Figure 4C**, filled circle). In addition, virtually no accumulation was observed for cellobiose (**Figure 4B**, open circle). This is probably because Ks5A7 has a higher catalytic efficiency in response to cellobiose than did Td2F2: Ks5A7, K_M_, 0.358 mM, and k_cat_, 155 s^-1^; Td2F2, K_M_; 4.44 mM, kcat; 7.13 s^-1^ (Uchiyama et al., 2013). Td2F2 is the GH1 BGL, which was obtained from the wood compost metagenome and is insensitive to glucose.

## Conflict of Interest Statement

The authors declare that the research was conducted in the absence of any commercial or financial relationships that could be construed as a potential conflict of interest.

## Footnotes

1 http://www.ncbi.nlm.nih.gov/BLAST/

2 http://www.afmb.cnrs-mrs.fr/CAZY/index.html

## Abbreviations

GH1: glycoside hydrolase family 1
pNP *p*-nitrophenol: pNPGlc *p*-nitrophenyl β-D-glucopyranoside
pNPFuc: *p*-nitrophenyl *β*-D-fucopyranoside
X-glc: 5-bromo-4-chloro-3-indolyl-β-D-glucoside
