# Erratum: Glucose-tolerant β-glucosidase retrieved from a Kusaya gravy metagenome

**DOI:** 10.3389/fmicb.2015.01131

**Published:** 2015-10-13

**Authors:** Frontiers Production Office

**Affiliations:** Frontiers Production Office, FrontiersLausanne, Switzerland

**Keywords:** β-glucosidase, cellulosic biomass, enzymatic saccharification, metagenome, substrate inhibition, product inhibition

Reason for Erratum:

Due to a typesetting error, the first author affiliation was listed incorrectly as Department of Medical Microbiology, University of Groningen, University Medical Center Groningen, Groningen, Netherlands.

The correct affiliation is: Bioproduction Research institute, National Institute of Advanced Industrial Science and Technology Tsukuba, Ibaraki, Japan.

The publisher apologizes for this mistake. This error does not change the scientific conclusions of the article in any way.

Original article was updated.

